# Research Progress on Dormancy Mechanism and Germination Technology of Kobresia Seeds

**DOI:** 10.3390/plants11233192

**Published:** 2022-11-22

**Authors:** Na Wang, Zhonghua Zhang, Wenhua Xu, Huakun Zhou, Rongchun Ning

**Affiliations:** 1 Northwest Institute of Plateau Biology, Chinese Academy of Sciences, Xining 810001, China; 2 Key Laboratory of the Cold Regions Restoration Ecology, Xining 810001, China; 3 University of Chinese Academy of Sciences, Beijing 100049, China

**Keywords:** germination rate, climate change, sustainability, alleviate dormancy

## Abstract

Kobresia is a subfamily of Cyperaceae, a perennial herbaceous plant that stores a large amount of organic carbon and nutrients (nitrogen, phosphorus, etc.) in the soil. This type of grass is soft and appreciated by all kinds of farm animals. It is one of the predominantly excellent fodder on the Qinghai–Tibet Plateau. Its good growth plays an important role in developing the local economy and maintaining ecological balance on the Qinghai–Tibet Plateau as well. The main objectives of this review are to systematically present and analyze the factors responsible for the low germination rate of Kobresia and to analyze the physical and chemical methods that are used in order to alleviate dormancy and to improve the germination rate of Kobresia seeds. This is performed in order to lay the foundation for future research in this field. At the same time, we have analyzed the research deficiencies and formulated recommendations for the future. This review will provide comprehensive information in order to reduce the cost of planting Kobresia, as well as to provide theoretical support and technical guidance for the purposes of ecosystem restoration and livestock development.

## 1. Introduction

Desertification is one of the most serious abiotic stresses currently facing humanity [1] and is associated with severe vegetation degradation and increased wind and water erosion [2]. In particular, desertification caused by wind erosion poses a serious threat to agricultural productivity and the ecological environment [3,4]. China is one of the countries that suffers the most from desertification in the world. In particular, the increasing aridity and desertification of grasslands in northwest China has led to serious ecological problems [5,6]. At the same time, due to natural and anthropogenic factors, the drying of wetlands has become a serious problem worldwide. As such, the restoration of tussock wetlands is urgently needed [7]. Wetland degradation can reduce the ability of regional ecosystems to self-regulate, leading to an increase in extreme environmental events, such as floods or droughts [8]. Previous studies suggest that tussock wetlands have a high potential to slow the increase in CO_2_ concentration due to global warming [9,10]. Vegetation plays an essential role in wetland creation, development, succession, and restoration [11,12]. It has also been found that 40 species of Kobresia are adapted to high moisture or subhumid climates [13]. As the climate warms, alpine grasslands are likely to deteriorate as permafrost decreases and glaciers melt [1]. Alpine vegetation on the QTP is at risk from global warming [14], partly due to the fact that the rate of warming on this plateau, in recent decades, has been twice the global average [15]. Under projected CO_2_ emission scenarios, global air temperatures are predicted to increase by 1.8–4.0 °C by 2100 [16]. Future warming could lead to significant changes in the community composition of alpine grasslands on the Tibetan Plateau. Species with higher germination rates and/or faster germination times will favor rapid space occupation and resource acquisition under the harsh living conditions that are found in alpine grasslands in a climate warming scenario. For example, Wang et al. [16] indicated that the germination percentage of *K. macrantha* increased and the average germination times were shortened in a climate warming scenario. Thus, the healthy growth of Kobresia plants plays an essential role in the establishment, development, succession, and restoration of wetlands [9].

Cyperaceae (sedges) represent the third largest monocot family in terms of species diversity, after Orchidaceae (orchids) and Poaceae (grasses), with 90 genera and 5500 species [17,18]. The family is distributed all over the world [19]. The first largest genus of Cyperaceae, the Carex Linn species, are distributed in various global climate zones [20]. Further, it is also one of the largest and most widely distributed genera in the world, with approximately 2000 species [21,22,23]. Kobresia is a subfamily of Cyperaceae. It is a cold mesophyte, wet mesophyte, and dry mesophyte; moreover, it is a perennial herb with dense clumps of underground buds with short rhizomes. It is distributed mainly in the temperate to cold regions of the Northern Hemisphere. It is a genus common in the northern temperate zone [24], commonly found at an altitude of 2000–5000 m, and is suitable for growing in alpine meadows. The long-term natural selection process makes Kobresia adapt to the alpine environment, and they can form dominant groups in alpine meadows as well [25]. Kobresia is the main vegetation type in the Qinghai–Tibet Plateau. As such, it, thus, plays an essential role in maintaining the plateau’s ecological balance [25,26].

Alpine meadows are the primary rangeland ecosystem types on the Qinghai–Tibetan Plateau (QTP). The Tibetan highlands encompass 83% of the Earth’s terrain above 4000 m and host the world’s largest pastoral alpine ecosystem [27]; as such, this region provides vast ecological and economic value. The Tibetan highlands are a center of Cyperaceae diversity, with >30 Kobresia species [27]. Kobresia is the main species of vegetation in the alpine region of the QTP. Moreover, the Kobresia meadow, with some species of this genus, is the main vegetation type in the QTP [25]. The Kobresia pastures of the Tibetan Plateau cover approximately 45 million hectares and constitute the world’s largest alpine pastoral ecosystems. These ecosystems are within the source region for the largest rivers in Southeast Asia and are mainly dominated by *K. pygmaea* [28,29]. However, these land cover types have suffered severe degradation at a regional scale [30], triggered by pronounced climate warming over the last several decades [31] and from progressively increasing anthropogenic disturbances, such as overgrazing [32,33].

Cold and humid climatic conditions are the characteristics of the QTP. In addition, the dwarf species with shallow roots prefer humid environments [34]. Kobresia is a perennial herbaceous plant that stores a large amount of soil organic carbon and nutrients (nitrogen, phosphorus, etc.). Further, the grass is soft and loved by all kinds of livestock. It is the main excellent forage in QTP. Its good growth plays an important role in developing the local economy and maintaining the ecological balance of the QTP [35,36].

Seed germination is a key ecological process that significantly affects subsequent seedling growth and plant development [37]. The completion of seed germination represents a key ecological and agronomic trait that determines when plants enter ecosystems [38]. Promoting seed germination and seedling growth is critical for the purposes of vegetation restoration. Moreover, a deep dormancy and low germination rate are the main bottlenecks for Kobresia seed germination and seedling growth, which restricts its nutritional and ecological value.

In recent years, much research has been carried out on seed dormancy and germination at home and abroad, certain results have been achieved by this [26,37,38,39,40,41], but there are still many problems to be solved. Domestic research on seed dormancy is still unsystematic; the research on the seed dormancy of Kobresia mainly focuses on the following: (1) Dormancy breaking is only treated by physical or chemical methods alone; (2) variable temperature is merely combined with other physical or chemical methods; (3) chemical methods for dormancy breaking are very limited, mainly using NaOH, GA_3_, and other reagents; and (4) at present, research on the dormancy and germination mechanism of Kobresia mainly focuses on physiological experiments. The molecular mechanism of seed dormancy is still not clear and how dormancy factors interact with each other is still unknown. There are few documents about the dormancy types of Kobresia seeds and the mechanism of improving the germination rate and different methods of breaking dormancy of different seeds. Therefore, a clear understanding of the scientific advances in the reasons for its low germination rate, as well as in the methods of relieving seed dormancy and increasing the germination rate of Kobresia are all very important for the design of future research on these species. However, a review of the current state of science, related to the causes of low germination rates and methods to alleviate seed dormancy and improve germination rates of Kobresia, is still lacking. Therefore, the main aims of this review are to systematically outline and analyze the factors responsible for the low germination rate of Kobresia, as well as to analyze physical and chemical methods in order to alleviate dormancy and improve the germination rate of Kobresia seeds. Thus, laying the foundation for future related research. At the same time, we have dissected the research deficiencies and will propose future recommendations. The solution to the above problems will provide theoretical support for the occurrence, evolution, adaptation, and development of Kobresia plants in the unique environment of the Qinghai–Tibet Plateau. Moreover, a further aim is to provide a basis for the rational utilization and protection of Kobresia plants in the future. At the same time, in this study, we will provide comprehensive information for the purposes of reducing the planting cost of Kobresia plants, as well as to provide technical guidance for ecosystem restoration and animal husbandry development [40,41].

## 2. Results

### 2.1. Reasons for the Low Germination Rate

Baskin and Baskin (2014) believed that seed dormancy is a phenomenon in which viable seeds cannot germinate under suitable conditions for a certain period of time [42]. The degree of dormancy reflects the environmental requirements for seed germination. Further, it also depends on the environmental requirements for germination (including temperature, moisture, and the magnitude of the environment required for germination (including temperature, moisture, light, oxygen, etc.)), which can also reflect the shallowness of seed dormancy [43]. Studies have found that the dormancy rate (ratio of the number of viable but non-germinating seeds to the total number of seeds tested, which is the same definition as the below) of Kobresia seeds collected below 2500 m was the highest, i.e., 87.9%; in addition, the seeds collected from 2500~3000 m were the lowest at 52.47%. Lastly, the average dormancy rate of the seeds collected from other altitude gradients was approximately 74% [44]. An important reason for the low germination rate of the seeds of the genus Kobresia is the high dormancy rate. The specific reasons for the high/low original germination rate (high/low) and the mechanism of germination rate change are shown in Table S1.

#### 2.1.1. Mechanical Obstruction of the Seed Coat

Seed coat structure determines seed viability and water absorption capacity. Studies have confirmed that the epidermal structure of seeds affects their germination [45,46]. It must be noted that dormancy is due to the impermeability of the seed coat to moisture and oxygen; further, this characteristic is called physical dormancy [47]. In order to adapt to the alpine ecological environment, the seeds of Kobresia have evolved continuously under environmental pressure and have, finally, developed mechanical protection tissues. It is precisely because of the blocking of mechanical tissues that it is difficult for the embryo to obtain oxygen and water from the outside world, thus limiting the germination of seeds. This is especially the case for the excessively thick structure at the base and top of the fruit, which limits the ability of the radicle and germ to penetrate the protective tissue [48]. The greater the thickness of the seed coat and its ratio to the caryopsis, the less likely the seed is to germinate [49]. Studies have shown that the pericarp of the seeds of Kobresia are thick, the appearance is dense, and the protective tissue is also extremely thick [50,51]. The pericarp and seed coat have poor air permeability and water permeability; moreover, the radicle and germ cannot penetrate the protective tissue [52]. Deng et al. [53] preliminarily researched the reproductive strategies of *T. tibialis* populations in alpine meadows and found that the indoor and field germination rates (i.e., the ratio of the number of germinating seeds during the test period to the total number of seeds tested, the same as below) of *T. chinensis* seeds were only 4% and 2%, respectively. The germination rates of seeds treated with sodium hydroxide (NaOH) solution and gibberellin (GA) solution were also determined. The seed germination rate was 47.3% after peeling off the seed coat, indicating that the hard seed coat was the main reason for the low seed germination rate. In a study of the alpine grass population, it was found that alpine grass is mainly vegetatively reproduced in alpine habitats. Although the seed yield is 4553.8 m^−2^, the germination rate indoors and in the field is only 4% and 1%, respectively. This is due to the hard seed coat, which is far less than the 52.6% seed germination rate that is found after peeling the seed coat [54].

#### 2.1.2. Existence of Endogenous Inhibitors

There are germination inhibitors in the seed coat, and this dormancy is commonly called chemical dormancy [52]. The seed coat or endosperm of many plant species contains certain phenols, aldehydes, and other chemicals that inhibit seed germination. Different seeds contain different inhibitory substances that are present in different parts of the seed structure, such as the coat, endosperm, or embryo. The germination process is influenced by three types of growth regulators: GA, which promotes germination; abscisic acid (ABA), which inhibits germination; and cytokinins (CK), which resist endogenous inhibitors. The germination process depends on the balance between these phytohormones. On this topic, Huang et al. [55] showed that a high content of endogenous ABA directly inhibits the germination of Kobresia seeds.

#### 2.1.3. Others

Some studies have found that there are a large amount of chromosome number and ploidy changes among the populations of the genus; in addition, it has also been found that the centromeres are scattered. Therefore, hybridization or polyploidy may have occurred among the genus—which affects seed fertility and which is one of the reasons for the low germination rate of Kobresia seeds [56].

### 2.2. Methods of Relieving Seed Dormancy and Increasing the Germination Rate of Kobresia

Breaking the dormancy of plant seeds usually depends on the factors mentioned below or due to a combination of them. This, also, further determines the strategy and diversity of plant species in plant communities.

#### 2.2.1. Physical Treatment

The first method that should be discussed is physical processing. This is especially the case for certain seeds with a poor permeable seed coat or with hard seeds. In addition, some physical and mechanical methods, such as cracking, rubbing, etc., are used to destroy the epidermis in order to release seed dormancy [52]. Yu et al. [57] found that the germination rate of broken bark seeds of dwarf grass in meadow soil and yak manure was significantly higher than that of intact seeds stratified in meadow soil and the control. Stratification made the broken seeds germinate more easily than the whole seeds. Moreover, *P. sturdy* has extensive dormancy. Kang et al. cut the seed coat by mechanical treatment and then treated it with chemical reagents and/or phytohormones, which can significantly promote the germination of *P. sturica* seeds [58].

The second method is temperature treatment. Hard seeds, or seeds that have not undergone physiological dormancy, can be effectively destroyed by variable temperature treatment. It must be noted that temperature plays an important role in regulating the germination of plant species [59]. Maximum soil temperatures that are greater than 50~55 °C were required for the purposes of hard-seed breakdown. Temperature changes can affect the germination time of plants [60,61], thereby determining the establishment and survival of seedlings in alpine ecosystems [62,63]. Rising temperatures can also accelerate seed metabolic responses during germination [43]. Seed germination is a complex process involving an ordered series of internal biochemical reactions, production, and elimination of substances and other processes (e.g., glycolysis and scavenging of a free radical), all of which need to be conducted at a specific temperature threshold, then reflected in the process of seed germination [64,65]. For example, higher temperature variation (12~22 °C) significantly increased the percentage for the germination of *K. schoenoides* [66]. Clarifying the effects of temperature on seed germination is crucial for predicting the changes in plant distribution, as well as community composition and renewal, which will also be useful for the purposes of sustainable management and the protection of natural ecosystems in the context of climate change.

The current study found that under the conditions of variable temperature, the germination rate of *K. macrantha* can be significantly improved [49]. Furthermore, the germination rate of *K. schoenoides* can achieve a breakthrough of 0~73.2% when changing the temperature in combination with cold stratification [66]. The germination rate of *K. robusta* Maximowicz was significantly improved by variable temperatures in combination with mechanical and chemical treatments [58].

The third option is cold stratification. Cold stratification can have positive, neutral, or negative effects on seed germination [67,68]. For seeds containing germination-inhibiting substances, the effect is obvious. On the one hand, stratification at low temperatures can gradually soften the seed coat and improve the permeability of the seed coat through moist conditions. On the other hand, the physiological values of seeds change during the stratification process. Small-molecule substances—which constitute a part of physiologically immature seeds—gradually become physiologically mature, such that seeds can enter the pregermination state and germinate when they encounter suitable environmental conditions [69]. For example, low-temperature treatment can stimulate the synthesis of endogenous gibberellins in seeds and the shoot tips of plants [70]). Zhao et al. [2] found that most seeds requiring low temperature stratification (i.e., 3~5 °C) for maturation have a problem with the balance and growth of endogenous hormones. In some plants, low-temperature treatment can achieve the same effect as exogenous gibberellin and thus stimulate seed germination [71,72]. In addition, cold stratification can replace the requirements of higher temperatures and light for germination in some species [73,74]. Moreover, wet-cold stratification has achieved good results in breaking the physiological dormancy of seeds of many plants in the Cyperaceae family [69,75]. Gou et al. [76] found that the longer the storage time in the refrigerator at 0~4 °C, the greater the conductance of the leachate decreased. In addition, the treatment with chemical reagents after 6 months in the refrigerator at 0~4 °C can significantly improve the germination rate of *K. myosuroides* (Villars) Foiri, *K. capillifolia*, and *K. prattii* seeds.

The fourth method is radiation treatment. This method is available with moderate X-rays, gamma rays, and ultrasound; moreover, it can also be treated with various illuminations [52]. Studies have shown that this method promotes seed germination. The combination of 12 h light/12 h darkness and variable temperature can significantly increase the germination rate of *K. schoenoides* and *K. macrantha* [49,66], specifically.

The physical methods for breaking the dormancy of Kobresia seeds are usually combined with other methods. The above physical methods have achieved good results in the laboratory. However, the positive effects on some species have not been confirmed in field trials.

#### 2.2.2. Chemical Treatment

One notable method of chemical treatment is conducting a treatment with exogenous plant hormones. Plant hormones play a versatile chemical role in seed germination. Further, plant hormones can respond to various physiological changes in seeds through signal transduction, regulate related enzymes, and protein metabolism, as well as control dormancy and seed germination [77]. Hormones mainly include fluridone (FL), gibberellin (GA), ethylene (ETH), cytokinin (CTK), kinetin, and melatonin (MLT). Due to plant germination inhibitors such as abscisic acid, plant seeds with a low germination rate can break and shorten dormancy via hormone treatment. For example, the exogenous application of GA_3_, which results in the activation of cytological enzymes and the enhancement of GA_3_ cytoderm plasticity and water absorption, altered the balance between high abscisic acid and low GA_3_ levels [78,79]. Moreover, FL hindered the synthesis of abscisic acid during seed germination, which contributed to reducing the content of abscisic acid in seeds. On the other hand, the ABA synthesis pathway was blocked, which indirectly promoted the synthesis of gibberellin in seeds. Further, CTK can abolish the dormancy caused by the inhibition of abscisic acid [80]. For example, when GA and ABA are simultaneously present, the germination-promoting effect of GA is inhibited; in addition, when GA, ABA, and CTK are simultaneously present, CTK can reverse this inhibition and allow germination, such that CTK is only necessary in the presence of ABA [77].

The use of hormones alone cannot effectively break dormancy and must be combined with other means, such as stratification at low temperatures, mechanical treatment to destroy the seed coat, treatment at variable temperatures, etc. For example, Huang et al. [55] found that the germination rates of *K. humilis* and *K. pygmaea* seeds were significantly increased under the combination of the plant hormone GA at the selected concentration and cold stratification for 30 days. The germination rates of *K. humilis* and *K. pygmaea* seeds increased the most at a concentration of 8.66 × 10^−4^ mol/L GA. They were 16.00%~41.00% and 12.67%~34.67%, respectively. Moreover, Li et al. [81] found that the optimal GA_3_ concentration to promote germination of *K. setchwanensis* was different at different temperatures. The optimal GA_3_ concentration were found to be 1.44 × 10^−4^ mol/Lat 20 °C. In addition, the optimal concentration was determined at 4.33 × 10^−4^ mol/L at 25 °C.

The second chemical treatment method is inorganic chemical treatment. Inorganic (e.g., strong acid, strong alkali, peroxide, bleach, etc.) reagents, such as potassium permanganate, hydrochloric acid, nitric acid, concentrated sulfuric acid, and peroxide, can effectively change the permeability of the seed coat and break the dormancy caused by the hard seed coat. For example, nitrate promotes germination by its binding NIN-like protein 8 to the CYP707A2 promoter and activating its expression, thereby reducing the levels of ABA after germination [82]. Saltpeter (KNO_3_) treatment can also improve seed quality, most likely due to the fact that K^+^ at an optimal concentration in KNO_3_ is used as a catalyst in order to increase the metabolic activity of adenosine triphosphatase (ATPase), nicotinamide adenine dinucleotide (NAD), and in promoting the biosynthesis and activity regulation of auxin in seeds [83].

It was found that experiments that are designed to improve the germination rate of Kobresia with sodium hydroxide (NaOH) treatment are very common. For example, in regard to the highest seed germination rate of *K. littledalei*, C. B. Clarke seeds were obtained via soaking seeds in 1.00 mol/L NaOH for 1 h [84]. The germination percentage of *K. humilis*, *K. capillifolia*, *K. macrantha*, and *K. prattii* was significantly increased by soaking seeds in 1.00 mol/L NaOH solution for 3 h [44]. Moreover, treatment with 1.00 mol/L NaOH + cold stratification can significantly improve the germination rate of *K. pygmaea* seeds, from 0 to 50.62%, which is the highest germination rate of *K. pygmaea* seeds in the existing studies [75]. Li QX et al. [81] found that the optimum concentration of NaOH was different at different culture temperatures. At 20 °C, the optimum concentration of NaOH treatment was 0.50 mol/L, and the germination rate of *K. setchwanensis* seeds increased from 76% to 96% [26]. In addition, the germination rates of *K. humilis* and *K. pygmaea* seeds were 16.33% to 56.00% and 13.33% to 46.00%, respectively. This was after soaking them in 10 mol/L NaOH for 1 h and storing them in a refrigerator at 4 °C for 30 days [75]. It must be noted that *K. robusta* Maximowicz had a comprehensive dormancy. Moreover, after 6 min of mechanical treatment (scoring the seed coat) and puncture treatment with concentrated sulfuric acid—as well as soaking with 8.66 × 10^−4^ mol/L GA_3_ for 48 h at 25/10 °C (12/12 h)—there was a significant promotion in the germination of *K.robusta* seeds. As a result, the germination rate increased from 27% to 64% [58]. Zhang et al. [66] found that in *K. schoenoides*, although stratification and its interaction with light and temperature fluctuations had strong effects on seed germination, *K. schoenoides* only germinated better under the combination of stratification, light, and higher temperature fluctuations than under other combinations of other factors. The changes in the seed germination rate of Kobresia under different treatments are shown in Table S2.

The factors affecting Kobresia seed germination are Interactive. Therefore, to break the dormancy of Kobresia seeds and to promote their germination, appropriate comprehensive measures must be taken according to the dormancy mechanism. In addition, there are no studies on treating Kobresia seeds with organic chemicals, such as polyethylene glycol, polyvinyl alcohol, thiourea, and acetone in order to break their dormancy and improve their germination rate.

## 3. Materials and Methods

### 3.1. Botanical Characterization and Distribution

#### 3.1.1. Botany

The genus Kobresia belongs to the sedge family and is a perennial herb. Approximately 80% of the world’s Kobresia plants are distributed in China. Furthermore, these plants are the dominant species of alpine vegetation in the world. The genus is divided into 3 groups, namely, the Kobresia group (Sect. Kobresia), the single-pointed Kobresia group (Sect. Elyna) and the isopointed Kobresia group (Sect. Hemicarex) [42]. Kobresia within China are mainly adapted to microthermal subhumid or mesothermal humid climate types [13].

#### 3.1.2. Plant Description

Perennial plants can be described in such terms as rhizome, short, creeping, upright, tufted, triangular, or cylindrical (with sparse or dense persistent leaf sheath at base). Moreover, leaves can be basal, less connate, flat, or margin curly linear. Most spikelets or single spikelets are terminal if the majority are composed of spike-like inflorescences or spike-like panicles. These can be bisexual or unisexual: where the latter of which is monoecious or heteroecious, which contains most spikelets. Spikelets can be unisexual or bisexual, where the unisexual type has only 1 male flower or 1 female flower, whereas bisexual types usually have 1 to several male flowers above one female flower at the base. Moreover, male flowers can be found with 1 scale and 2~3 stamens in the axillary. Female flowers can also have 1 scale, as well as 1 pre-emerged leaf formed by the healing of two bracts in axillary, opposite scales, and 1 pistil wrapped by a pre-emerged leaf. In addition, female types can possess upper ovaries, 2~3 stigma fruits (which are small nuts, triangular, biconvex, or flat convex and are completely or incompletely wrapped by first leaves). It must also be noted that a degraded spikelet axis usually exists in female branch spikelets.

#### 3.1.3. Geographical Distribution

Plants of the genus Kobresia are distributed at altitudes of 3100~5300 m, 81~112° E, and 23~46° N in longitude and latitude, respectively. However, *K. pygmae* can be found at the lowest altitude of 1100 m in Nepal and *K. duthiei* can live at an altitude of 5700 m. The main growing areas of the genus include slopes, lakeshores, valleys, alluvial fans, floodplains, and scrub under shrubs [13,25]. They are mainly distributed in the temperate to cold regions of the Northern Hemisphere, with most of them concentrated in the Himalayas and the Hengduan Mountains, where they are particularly suitable for various habitats in the alpine region. The Himalayas are the main center of distribution and the birthplace of the flora [85]. In China, the genus Kobresia is distributed in the QTP and in the northwestern, northern, and northeastern parts of China, where 13 species are widely distributed and 10 intermittently [13]. The global distribution and growth environment of Kobresia in China are listed in Table S3.

#### 3.1.4. Modes of Reproduction

The plants of the genus Kobresia are mainly dominated by vegetative reproduction via underground short rhizomes and secondary sexual reproduction. The vegetative reproduction efficiency accounts for 90.92% of the total reproduction efficiency [53]. At the same time, the study found that the ability of the genus Kobresia to produce seeds is not small. Deng et al. [53] conducted a study on the population reproduction strategy of dwarf grass in an alpine meadow and found that the seed production capacity was 715.5 grains m^−2^. The seeds were basically mature at the end of the growing season, the indoor seed germination rate of the year reached 88.6%, and the seed germination rate of the seed bank was approximately 70% [53]. However, due to the harsh environment of the QTP and the hard seed coat, the germination rate under field conditions is only 3%; further, the proportion of seeds that can finally germinate into seedlings is relatively small, as a result [53,54].

### 3.2. Methods

#### 3.2.1. Search Strategy

The publications included in this study were searched in the Elsevier (https://www.sciencedirect.com/, accessed on 27 July 2022.), ACS (https://pubs.acs.org/, accessed on 27 July 2022.), Springer (https://link.springer.com/, accessed on 27 July 2022.), Web of Science (https://www.webofscience.com/wos/alldb/basic-search, accessed on 27 July 2022.), PubMed (https://pubmed.ncbi.nlm.nih.gov/, accessed on 27 July 2022.), Google Scholar (https://scholar.google.com.br/, accessed on 27 July 2022.), Baidu Scholar (https://xueshu.baidu.com/, accessed on 27 July 2022.), Wiley (http://qikan.cqvip.com/, accessed on 27 July 2022.), and China National Knowledge Infrastructure (CNKI) (https://www.cnki.net/, accessed on 27 July 2022.) databases. The keywords used in the search were “Cyperaceae”, “Kobresia”, “Germination”, and “Dormancy”.

#### 3.2.2. Study Selection

The inclusion criteria were review articles and research articles addressing specific information about new species, botany, geographic distribution, methods of germination and the dormancy types of Kobresia published in the last 21 years (2000–2021). The exclusion criteria were e-books, book chapters, and conference abstracts.

#### 3.2.3. Data Extraction

A total of 346 scientific documents were initially selected. Among them, the titles and abstracts of 241 documents that did not reflect the focus of this review were excluded. Finally, we analyzed and screened 105 articles according to the specific information in the title, abstract, and full text.

## 4. Conclusions and Future Prospective Developments

This review found that the high dormancy of the Kobresia species has the greatest effect on their low germination rate. This is mainly because of the hard and thick seed coat, the fact that the base and top of the seed coat are relatively thick, and due to the thickness of the protective tissue, which accounts for only a small proportion of the thickness of the fruit cross-section. Another reason that should be noted are the endogenous inhibitors in the seeds that lead to the low germination rate of Kobresia seeds. All of these factors indicate that the dormancy of Kobresia seeds is caused by many factors and that more than two methods are required in order to break dormancy. The dormancy type and degree of dormancy of seeds of different Kobresia species were different. However, for the same Kobresia species, the germination rate was different at different collection sites. In the context of the same treatment, the germination rate of the same species increased or decreased at different collection sites. The optimal concentration of the NaOH treatment for the same species was different at different culture temperatures. In general, the difference in germination can be attributed to the interaction between genetic genes and environmental conditions. Therefore, different species require different pathways in order to release dormancy, and it is necessary to study different standard germination tests. In further experimental research, on the one hand, it is necessary to focus on the combination of physical and chemical methods in order to solve the physical dormancy of seeds first. Then, the chemical dormancy of seeds should be looked at next, that is, to treat the limitation of the seed coat first and then solve the chemical dormancy of seeds by chemical methods, such as through plant hormones, and to thus increase the effect of plant hormones (such as cytokinin, auxin, and ethephon) on the germination of Kobresia seeds. At the same time, it is necessary to comprehensively analyze the different effects of chemical reagents and various plant hormones on the internal and external effects of seeds and design experiments.

On the other hand, further experimental studies require habitat data in order to support plant growth, such that experimental results can better reflect natural conditions. At the same time, the method of improving the seed germination rate in the laboratory is applied to field experiments in order to observe whether the change trend of the germination rate in the laboratory matches that in the field in the context of providing technical guidance for the purposes of field cultivation and vegetation restoration. Future research should focus on the regulatory mechanism of seed dormancy and germination, the relationship between metabolism, as well as seed dormancy and key processes. By identifying more genes and expressing proteins, we further evaluated the functions of specific genes and proteins in the germination process. On this basis, the interaction between proteins during seed germination will be studied in depth. In addition, the actual level of protein seed dormancy and germination research will be explored. With the help of various new technologies of genomics, transcriptomics, and proteomics, the mechanism of seed dormancy and dormancy breaking is explored at the overall level in order to find more species with higher germination rates and/or faster germination times under climate warming scenarios. From another point of view, the micromorphological structure of seeds determines the water absorption and viability of seeds and is also closely related to seed diffusion, longevity, and germination. In addition, sexual reproduction is more important than asexual reproduction for the sustainable development and regeneration of a population. Therefore, the germination ecology of Kobresia seeds after maturation is a theoretical problem worthy of further study.

In this article, we systematically summarized the reasons for the low germination rate of Kobresia. These include the mechanical obstruction of the seed coat and the presence of inhibitors, as well as the physical and chemical methods in order to relieve seed dormancy and to increase the germination rate of Kobresia—which can aid in a faster and more efficient process in the development of dormancy mechanisms and germination technology for Kobresia seeds.

## Data Availability

The data presented in this study are available on request from the corresponding authors. The data are not publicly available due to privacy.

## Supplementary Materials

The following supporting information can be downloaded at https://www.mdpi.com/article/10.3390/plants11233192/s1. Table S1: Seed Germination Mechanism of Kobresia; Table S2: Germination of seeds of Kobresia under different treatments; and Table S3: Distribution and habitat of the species of the Kobresia genus in China. Refs. [13,16,24,26,31,49,55,57,75,76,84,86,87,88,89,90] have been cited in supplementary materials.
