# Supplementary material for: Research Progress on Dormancy Mechanism and Germination Technology of Kobresia Seeds
Source: Plants (Basel). 2022 Nov 22;11(23):3192. doi: 10.3390/plants11233192 (PMC9736853; doi:10.3390/plants11233192)
Supplement: Supplementary file 1 [file plants-11-03192-s001.zip › plants-1990677-supplementary.pdf]

Table S1. Seed Germination Mechanism of Kobresia

NO.	Species	Reasons for High / Low Original Germination Rate and Dormancy Type	Causes of Germination Rate Change (Mechanism)	Reference
1	*K. httledalei* C.B. Clarke	1, Dormancy is evident; 2, A hard, thick seed coat, the base and top of the seed coat are thick, and the thickness of the protective tissue is unusually large in relation to the thickness of the fruit cross-section.<br>Physiological dormancy	1, NaOH strong alkali damage, seed coat corrosion, change the permeability, eliminate the resistance of the seed coat, so that the radicle can easily break through the seed coat; 2, Effectively reduce the content of seed coat inhibitor-endogenous abscisic acid in Cobresia seeds; 3, Destroy the cuticle of seeds, to increase the intercellular space of the seed coat, which promotes the material exchange between Artemisia seeds and the outside; 4, Low temperature stratification treatment can also play a role in breaking the physiological dormancy of Kobresia seeds and reducing the endogenous abscisic acid.	[49, 75, 84]

2	*K*．*Humilis*	1, The main reason of low germination rate is that the seed coat is thick, hard and compact, covered with smooth and thick cuticle; 2, the base and top of seed coat are thick, the ratio of the thickness of protective tissue to the thickness of fruit transverse section is rare; 3, the content of endogenous ABA is high.	1, The dormancy properties of broken seed can be relieved, it germinates more easily; 2, Stratification makes broken seeds germinate more easily, which is equivalent to the germination rate of broken seed excreted from the animal's digestive tract after natural stratification in feces or soil; 3, Seeds treated with a certain concentration of NaOH solution can uniformly remove the cuticle of the shell, and even destroy the outermost large thick-walled cells of the shell, increase the intercellular space, loosen the middle elongated dense tissue, and facilitate the entry of water and oxygen; 4, The endogenous ABA content of *K. humilis* seeds was still very high after NaOH treatment, although much lower than before treatment. The endogenous ABA content of *K. humilis* and *K. pygmaea* was 1.225 μg / g and 1.813 μg / g, respectively.	[16, 55, 57]
3	*K*．*royleana*	1. The germination rate of *K*．*royleana* seeds without any treatment reached 91%, which was related to the fact that compared with other Kobresia seeds, the outermost epidermal cell wall of Kobresia himalayana seeds was thinner, with slits and less wax (observed under anatomical microscope), which was relatively beneficial to the entry of water and oxygen and the penetration of embryo. 2, No endogenous ABA detected in seeds.	1, A certain concentration of NaOH solution in the treatment of seeds can uniformly remove the cuticle of the shell, destroy even the outermost large thick-walled cells of the shell, increase the intercellular space, loosen the middle elongated dense tissue, and facilitate the penetration of water and oxygen; 2, ABA was not detected in the seeds of *K. himalayana* in the control group and the seeds treated with NaOH.	[16, 55, 75]

4	*K. pygmaea*	1, The base and upper surface of the seed coat are thick, hard and dense; 2, Smooth and thick cuticle; 3, High endogenous content ABA.	1, NaOH solution treatment can destroy the seed cuticle, increase the cell gap of the seed coat, which promotes material exchange between the seed and the outside; 2, NaOH solution treatment of Artemisia seeds can effectively reduce the content of seed coat inhibitors and endogenous abscisic acid in Kobresia seeds to 1.814 μg /g; 3, NaOH solution treatment significantly increases the activity of peroxidase, superoxide dismutase and dehydrogenase in Kobresia seeds; 4, Cold stratification treatment also has a certain effect on breaking physiological dormancy of Kobresia seeds and reducing endogenous abscisic acid.	[16, 55, 75]
5	*K. capillifolia*	1, The seed coat is hard and dense, smooth and thick cuticle; 2, The seed coat base and top are relatively thick, and the thickness of the protective tissue cross-section of the fruit thickness ratio is too large.	1, After NaOH treatment, the seed coat of Kobresia was ruptured, damaged, corroded and softened to different degrees, so that the permeability of the seed coat was improved and the germination rate was increased; 2, After prolonged storage, part of the dormancy can be lifted, the germination rate increases; the longer the storage time, the more the conductivity of the leaching solution decreases.	[16, 76]
6	*K. macrantha*	1, The seed coat is hard and dense, smooth and thick cuticle; 2, The seed coat base and top are relatively thick, and the thickness of the protective tissue cross-section of the fruit thickness ratio is too large.	1, After NaOH treatment, the seed coat of Kobresia pygmaea was torn, damaged, corroded and softened to different degrees, so that the permeability of the seed coat was improved and the germination rate was increased; 2, The activity of peroxidase, superoxide dismutase and dehydrogenase in Kobresia seeds significantly increased; 3， Increasing temperatures can accelerate the metabolic responses of seeds during germination.	[16, 49]

7	*K． prattii*	1, The seed coat is hard and dense, smooth and thick cuticle; 2, The seed coat base and top are relatively thick, and the thickness of the protective tissue cross-section of the fruit thickness ratio is too large.	1, After NaOH treatment, the seed coat of Kobresia songarica seeds was broken, damaged, corroded and softened to different degrees, so that the permeability of the seed coat was improved and the germination rate was increased. 2, After a certain period of storage, dormancy can be gradually released; 3, the longer the storage time, the more the conductivity of the leaching solution decreases.	[16, 49, 76]
8	*K. setchwanensis*	Complete hard seed coat directly hinders the water absorption and oxygen through the seeds, reducing the seed germination rate and germination rate.	1, The seed coat was softened and is no longer a limiting factor for seed germination after being soaked in an appropriate concentration of NaOH; 2, Under different temperature conditions, the ability of Kobresia seeds to absorb exogenous chemical reagents and synthesize endogenous gibberellins was different; 3, GA_3 and $CaCl_2$ are germination-promoting substances. Treatment with GA_3 solution in appropriate concentration can improve seed vigor and promote seed germination.	[26]
9	*K. robusta Maximowicz*	Seed structure hard, weak seed coat permeability and permeability, resulting in the growth and development of embryos can not get enough water and oxygen, thus inhibiting seed germination.	1, Treatment with concentrated H_2SO_4 improved the water and air permeability of the seed coat and reduced the seedling elongation limitation caused by the thick seed coat; 2, Variable temperature treatment softened the seed coat; 3. GA_3 broke dormancy caused by the presence of germination inhibitors in the seed coat.	[24, 31, 49]
		Combinational dormancy		
10	*K. setchwanensis*	Hard dense protective structure.	NaOH destroyed the seed coat structure	[26]
11	*K. myosuroides* (Villars) Foiri	--	The longer the storage time, the more the conductivity of leaching solution decreases	[76]

12	*K. schoenoides*	--	For the stratified seeds, higher temperature fluctuation decreased germination percentage in darkness, and increased germination percentage in light	[75]

Table S2. Germination of seeds of Kobresia under different treatments

NO.	Species	Collection sites	Germination Treatment Method	Germination Rate Change	Reference
1	*K. httledalei* C.B. Clarke	Tibet Naqu	Soak seeds in 30g/L NaOH for 1h	43.00%~96.00%	[49, 75, 84]
		Tibet Naqu	Soak seeds in 40g/L NaOH for 1h	43.00%~98.00%	
		Tibet Naqu	Soak seeds in 40g/L NaOH + low temperature stratification 30d	Significantly higher than those in control group	
		Tibet Langkazi	Soak seeds in 40g/L NaOH for 1h + low temperature stratification 30d	70.50%~86.00%	
		Tibet Dangxiong	Soak seeds in 40g/L NaOH for 3h + low temperature stratification 30d	72.00%~82.00%	
2	*K．Humilis*	Zhuaxi Xiulong Township, Tianzhu Tibetan Autonomous County, Wuwei City, Gansu Province	Meadow soil middle layer, yak dung middle layer + scratched seed coat	Significantly higher than those in control group	[16, 55, 57]
		Tibet Dangxiong	Soak seeds in 40g/L NaOH for 3h	21.00%~63.00%	
		Tibet Dangxiong	Soak seeds in 40g/L NaOH for 1h + low temperature stratification 30d	16.33%~56.00%	
		Tibet Dangxiong	Soak seeds in 8.66×10^{-4}mol/L GA for 1h+ stratification at 4 °C for 30 d	12.67%~34.67%	
		Tianzhu County, Gansu Province, Southwest Wushaoling Foot	Soak seeds in 4% NaOH for 3h	27.3%~86.7%	
		Tianzhu County, Gansu Province, Southwest Wushaoling Foot	Soak seeds in 5% NaOH for 3h	27.3%~81.3%	
		Tibet Naqu	Soak seeds in 40% NaOH for 3h + low temperature stratification 30d	12.5%~40.7%	
3	*K．royleana*	Tibet Langkazi	Soak seeds in 40 g/L NaOH for 3h	91.00%~96.00%	[16, 49, 55]
		Tibet Langkazi	Soak seeds in 40% NaOH for 1h + low temperature stratification 30d	91.33%~94.00%	
		Tibet Langkazi	Soak seeds in 40% NaOH for 3h + low temperature stratification 30d	91.0%~94.0%	

		Tibet Naqu	Soak seeds in 40% NaOH for 3h + low temperature stratification 30d	75.0%~83.0%	
4	*K*． *pygmaea*	Tibet Naqu	Soak seeds in 40g/L NaOH for 3h	0~50.00%	[16, 55, 75]
		Tibet Naqu	Soak seeds in 40g/L NaOH + low temperature stratification 30d	0~50.62%	
		Tibet Naqu	Soak seeds in 8.66×10^{-4}mol/L GA for 1h+ stratification at 4°C for 30 d	16.00%~41.00%	
		Tibet Naqu	Soak seeds in 40% NaOH for 1h + low temperature stratification 30d	13.33%~46.00%	
		Tibet Naqu	Soak seeds in 40% NaOH for 3h + low temperature stratification 40d	0~43.90%	
5	*K*． *capillifolia*	Tibet Dangxiong	Soak seeds in 40% NaOH for 3h	14.00%~74.00%	[16, 76]
		Tibet Dangxiong	Soak seeds in 40% NaOH for 3h + low temperature stratification 30d	12.50%~70.00%	
		Tianzhu County, Gansu Province, Southwest Wushaoling Foot	Soak seeds in 5% NaOH for 3h<br>Soak seeds in 5% NaOH for 4h	0~18.00%<br>0~16.00%	
		Laji Mountain, Guide County, Qinghai Province	Store in refrigerator at 0-4°C for 6 months+Soak seeds in 20 g/L NaOH for 3h	0~37.50%	
6	*K*． *macrantha*	Tibet Langkazi	Soak seeds in 40 g/L NaOH for 3h	0~90.00%	[16, 49]
		Tibet Langkazi	Soak seeds in 40% NaOH for 3h + low temperature stratification 30d	1.00%~89.30%	
		Damxung Grassland Station located in the southern foothills of Nyenchen Tanglha	12h light/12h dark; Variable temperature: 25°C /15°C	about10.00%~35.00%	
7	*K*． *prattii*	Tibet Dangxiong	Soak seeds in 40%NaOH for 3h	69.00%~96.00%	[16, 49, 76]
		Tibet Dangxiong	Soak seeds in 40%NaOH for 3h + low temperature stratification 30d	61.50%~88.70%	
		Tongren County, Huangnan Prefecture, Qinghai Province	Store in refrigerator at 0-4°C for 6 months + Soak seeds in 40 g/L NaOH for 3h	0~37.50%	
8	*K. setchwanensis*	Maqu County, Gannan Tibetan Autonomous Prefecture, eastern Qinghai-Tibet Plateau	The optimum concentration of NaOH is 30 g/L at 10°C	8.00%~30.00%	[26]

		Maqu County, Gannan Tibetan Autonomous Prefecture, eastern Qinghai-Tibet Plateau	The optimum concentration of NaOH is 20 g/L at 20°C	76.00%~96.00%	
		Maqu County, Gannan Tibetan Autonomous Prefecture, eastern Qinghai-Tibet Plateau	Under the condition of GA_3, the optimum treatment concentration was 0.05 g /L at 20 °C; the optimum treatment concentration was 0.15 g /L at 25°C.	76.00%~80.00%； 56.00%~60.00%	
		Maqu County, Gannan Tibetan Autonomous Prefecture, eastern Qinghai-Tibet Plateau	Under the condition of $CaCl_2$, the optimum treatment concentration was 5 g /L at 10°C; the optimum treatment concentration was 10 g/L at 20°C; the optimum treatment concentration was 15 g /L at 25°C.	8.00%~24.00%;76.00%~80.00%;56.00%~76.00%	
9	*K. robusta Maximowicz*	Ouqiang Village, Oula Township, Maqu County, Gansu Province	Soaking seeds with 300 mg/ L GA_3 for 48 h at 25/10°C (12/12 h) after mechanical treatment (breaking the seed coat) and puncture treatment with concentrated sulfuric acid for 6 min was able to significantly promote seed germination of *K. robusta Maximowicz*	27.00%~64.00%	[31]
10	*K. setchwanensis*	Tibet Naqu	Soak seeds in 40% NaOH for 2h	92.00%~98.00%	[26]
11	*K. myosuroides* (Villars) Foiri	Tongren County, Huangnan Prefecture, Qinghai Province	Store in refrigerator at 0-4 °C for 6 months + Soak seeds in 40 g/L NaOH for 3h	0~17.50%	[76]
12	*K. schoenoides*	Qinghai Haibei Alpine Meadow Ecosysytem Research Station	12h light/12h dark; Cold stratification at 4°C for 30 days; 12-22°C variable temperature.	0~73.20%	[75]

Table S3. Distribution and habitat of the species of the Kobresia genus in China

NO	Species	Distribution Area	Habitat	References
1	*K. pygmaea* C. B. Clarke	Distribution in China: Inner Mongolia, Hebei, Shanxi, Gansu, Qinghai, southern Xinjiang, Sichuan, Yunnan, Tibet;<br>Overseas distribution: Bhutan, Nepal to Kashmir	Alpine shrub meadows and alpine meadows at an altitude of 3200-5400 m	[86]
2	*K.humilis(C.A.Mey.)Serg*	Distribution in China: Xinjiang, Tianshan Mountains, Altai Mountains, Bole and Hot Spring Mountains;<br>Overseas distribution: Kazakhstan, Uzbekistan, Kyrgyzstan, Tajikistan, Turkmenistan	Alpine meadows, swampy meadows and shrub meadows at an altitude of 2000-3400 m	[86]
3	*K. peach*	Distribution in China: Tibet, Xinjiang;<br>Overseas distribution: Sikkim, Nepal	--	[87, 88]
4	*K. prainii*	Distribution in China: Tibet;<br>Overseas distribution: Bhutan	Alpine shrub meadows and alpine meadows at an altitude of 4100-5300 m	[86]
5	*K. littledalei* C.B.Clarke	Distribution in China: Southern Xinjiang, China (Tashkorgan)	Alpine meadows and swampy meadows at an altitude of 4200-5400 m	[89]
6	*K. deasyi* C.B.Clarke	Distribution in China: Xinjiang (Kunlun Mountains and Pamirs), northwestern to southern Tibet;<br>Overseas distribution: Nepal, Afghanistan, Kyrgyzstan (Tianshan), Tajikistan (Pamir)	Alpine meadows, swampy meadows and thickets at an altitude of 3800-5000 m	[86]
7	*K. schoenoides*	Distribution in China: Xinjiang (Altai Mountains), western Sichuan, northwestern Yunnan, eastern Tibet;<br>Overseas distribution: Bhutan, Nepal, Kashmir, Kazakhstan (Central Asia), Georgia (Caucasus), Russia (Siberia);	Hillside meadow at an altitude of 2500-3500 m	[86]
8	*K. robustaMaxim*	Distribution in China: Gansu, Qinghai, Tibet;<br>Overseas distribution: Kunlun Mountains, Pamirs	Sandy soil at an altitude of 4000-4600(4700) m	[88]

9	*K. macranthaBoeck*	Distribution in China: Western Sichuan, Gansu, Qinghai, Tibet; Overseas distribution: Nepal	Alpine meadows, lakeside and ditchside meadows at an altitude of 3600-4700 m	[86]
10	*K. daqingshanica*	Distribution in China: Inner Mongolia	Hillside meadow at an altitude of 1800-2000 m	[13]
11	*K. curticeps*	Distribution in China: Southern Tibet;<br>Overseas distribution: Sikkim, Nepal	Woodside Meadow at an altitude of 3000-3600 m	[13]
12	*K. Vidua*	Distribution in China: southeastern Gansu, southwestern Qinghai, western and southwestern Sichuan;<br>Overseas distribution: Nepal	Moist grassland, swamp meadow and alpine shrub meadow at an altitude of 3000-5100 m	[86]
13	*K. glaucifolia*	Distribution in China: southern Xinjiang (Tashkorgan) and southern Tibet	Meteralpine scrub meadows and swamp meadows at an altitude of 3600-4800 m	[86]
14	*K. kansuensis*	Distribution in China: eastern Gansu, southern Qinghai, western and southwestern Sichuan, northwestern Yunnan, eastern Tibet	Alpine thickets, floodplains, wet grasslands, shady hillsides and forest-edge grasslands at an altitude of 3000-4800 m	[90]
15	*K. pusilla*	Distribution in China: Inner Mongolia, Hebei, Gansu, Qinghai, western Sichuan, Tibet	Alpine meadow and swamp meadow at an altitude of 3200-5300 m	[86]
16	*K. williamsii*	Distribution in China: Tibet;<br>Overseas distribution：Nepal	Under the fir forest at an altitude of 3800 m	[86]
17	*K. uncinoides*	Distribution in China: western and southwestern Sichuan, northwestern Yunnan, Tibet;<br>Overseas distribution: Bhutan, Sikkim, Nepal	Alpine shrub meadows, swampy meadows, riverbank meadows and forest-side meadows at an altitude of 3400-4500 m	[86]
18	*K.graminifolia* C.B.Carke	Distribution in China: Shaanxi (Taibai Mountain) and southern Gansu	The top of the mountain, the crevice of the rock or in the forest grass at an altitude of 3100-3800 m	[13]
19	*K. helanshanica*	Distribution in China: Gansu, Qinghai, Sichuan, Yunnan, Tibet	Hillside meadow at an altitude of 2900 m	[13]
20	*K. loliacea*	Distribution in China: southwestern Sichuan, northwestern Yunnan, eastern Tibet Autonomous Region	Limestone hillside meadows, hilltop meadows, alpine oak or spruce forest margins	[13]
21	*K. lacustris*	Distribution in China: Southwest Sichuan	Gravel on the shore of a alpine glacier lake at an altitude of 4450 m	[13]

22	*K.Curticepsvargyirongensis* Y.C.Yang	Distribution in China: Tibet: Yadong	Underwood edge	[13]
23	*K. seticulmis*	Distribution in China: southwestern Sichuan, western and northwestern Yunnan, southeastern Tibet;<br>Overseas distribution: Northern India, Sikkim, Nepal	Alpine scrub meadow with rocks at an altitude of 3600-4300 m	[13]
24	*K. sister-in-law*	Distribution in China: Gansu, Qinghai, Sichuan, Songnan, Tibet	Alpine shrub meadows, swamps in alpine meadow belts, forest meadows, or wet hillsides at an altitude of 3000-4750 m	[13]
25	*K. filicina*	Distribution in China: Northwestern Yunnan and eastern Tibet; Overseas distribution: Northern India	Under the forest or rocks by the river at an altitude of 2900-4000 m	[90]
26	*K.jaw*	Distribution in China: Western Sichuan	Subalpine and alpine meadow belt at an altitude of 2500-3400 m	[86]
27	*K. nitens*	Distribution in China: Western Tibet;<br>Overseas distribution: Western Nepal to Kashmir	--	[90]
28	*K. lepidochlamys*	Distribution in China: Northwest Yunnan	Hillside meadow at an altitude of 4300 m	[13]
]29	*K. maquensis*	Distribution in China: Southern Gansu and northwestern Sichuan	Alpine meadows at an altitude of 3500 m	[13]
30	*K. menyuanica*	Distribution in China: Qinghai(Menyuan County)	Alpine shrub meadow at an altitude of 3500 m	[90]
31	*K. minshanica*	Distribution in China: Southeastern Gansu (Minxian), western Sichuan (Ganzi) and southwestern (Muli Tibetan Autonomous County)	Alpine shrub meadow at an altitude of 3800 m	[86]
32	*K. fragilis*	Distribution in China: Western and southwestern Sichuan Province, northwestern Yunnan Province;<br>Overseas distribution: eastern Nepal, Tibet Autonomous Region	The edge of evergreen broad-leaved forest, under alpine oak forest and alpine shrub meadow at an altitude of 2700-4500 m	[90]
33	*K.nepalensis (Nees)* Kukenth	Distribution in China: Sichuan, Yunnan, Tibet;<br>Overseas distribution: Nepal, Sikkim	Alpine shrub meadows and alpine meadows or rocky beaches at an altitude of 3600-4600 m	[88]
34	*K. kuekenthaliana*	Distribution in China: Southwest Sichuan (Xichang area), northeast Yunnan	Hillside meadow at an altitude of 2700 m	[86, 90]
35	*K. inflate*	Distribution in China: northwestern Yunnan, southeastern Tibet	Alpine meadows at an altitude of 4500-4600 m	[13]
36	*K. stolonifera*	Distribution in China: Southern Xinjiang (Pishan), Western and Northern Tibet	Dunes, river valley sand, gravel and lake meadows at an altitude of 4500-5300 m	[13, 90]

37	*K. burangensis*	Distribution in China: Western Tibet	Alpine meadow at an altitude of 5000 m	[86]
38	*K. macroprophylla*	Distribution in China: Western Gansu (Sunan County), northeastern Qinghai (Qilian, Menyuan, Ledu)	Sunny slopes, semi-shady slopes and foothill meadows at an altitude of 2400-2800 m	[86, 87]
39	*K.prainii Kük*	Distribution in China: Tibet: Zhongba, Saga, Kangma, Gyantse and Qushui counties	Hillside grass and hillside gravel at an altitude of 3900-5600 m	[86]
40	*K.esanbeckii*	Distribution in China: northwestern Yunnan, southern Tibet, Sikkim, Nepal	Alpine oak on the rocks under the forest at an altitude of 3200-3400 m	[13]
41	*K. laxa*	Distribution in China: Kashmir, southern Tibet, northern India, Nepal, Sikkim	The side of the ditch under the hemlock forest at an altitude of 2600-2700 m	[13]
42	*K. filifolia*	Distribution in China: Inner Mongolia, Hebei, Shanxi, Gansu Russia (Siberia)	Hillside meadow at an altitude of 1700-2750 m	[13]
43	*K. setchwanensis*	Distribution in China: Qinghai (Dawu Township in Maqin, Delerong, Baiyu Township in Jiuzhi, Kesuogou, Kangsai Township, and Makehe Forest Farm in Banma), Gansu (Oula Township, Nima Township, Hequ Army in Maqu) Horse farms), eastern Tibet, western and northern Sichuan, and northwestern Yunnan	Alpine and subalpine belts, forest margins and glades at an altitude of 3600-4300 m	[13]
44	*K. pinetorum*	Distribution in China: Northwest Yunnan Province	Hot and dry valley hillside Yunnan pine forest at an altitude of 2600 m	[86]
45	*K. myosuroides*	Distribution in China: Heilongjiang, Jilin, Inner Mongolia, Hebei, Shanxi, Gansu, Qinghai, Xinjiang, Sichuan, Yunnan, Tibet; Overseas distribution: Russia (Siberia, Far East), Kazakhstan (Central Asia), Kyrgyzstan (Tianshan), North Korea, Japan, Mongolia, Europe, North America	Floodplains, wet grasslands, understory, swamp meadows and scrub meadows at an altitude of 2600-4800 m	[86]
46	*K. caricina*	Distribution in China: Western Tibet;<br>Overseas distribution: Europe, North America, Georgia (Caucasus), Kazakhstan (Central Asia), Western Himalayas to Nepal	Swampy meadows and floodplains at an altitude of 3500-4600 m	[90]

47	*K. thewhore*	Distribution in China: Southern Tibet Autonomous Region (Yadong County);<br>Overseas distribution: Sikkim	Sandy hillside at an altitude of 3700 m	[86]
48	*K. cercostachya*	Distribution in China: Sichuan, Yunnan and Tibet, the Qinghai-Tibet Plateau	On gravel or edge of rocky beach in alpine shrub-meadow belt at an altitude of 3600-5000 m	[90]
49	*K. Tibetan*	Distribution in China: Gansu, Qinghai, western Sichuan, eastern Tibet	Floodplains, moist grasslands, alpine shrub meadows at an altitude of 3000-4600 m	[86, 90]
50	*K.royleana* (Nees) Boeckeler	Distribution in China: Qinghai, western and southwestern Sichuan, northwestern Yunnan, Tibet;<br>Overseas distribution: Nepal, northern India, Afghanistan, Tajikistan, Kazakhstan	Alpine meadow, alpine shrub meadow, swamp meadow, river floodplain, etc. at an altitude of 3100-5200 m	[86, 87]
51	*K. stenocarpa*	Distribution in China: Central Asia and Xinjiang, Tibet and Gansu in mainland China	Wet meadows, floodplains or hillsides at an altitude of 2600-4600 m	[13, 86, 87]
52	*K. narrow*	Distribution in China: Northwest Yunnan and Eastern and Southern Tibet;<br>Overseas distribution: Sikkim	Alpine shrub meadows and rocks under the fir forest at an altitude of 3200-4000 m	[86]
53	*K. squamaeformis*	Distribution in China: Southern Gansu (Xiahe County)	Alpine meadows at an altitude of 3300-3600 m	[13, 86, 87]
54	*K. yangii*	Distribution in China: Southwest Sichuan	Alpine oak forest meadow at an altitude of 3600-4400 m	[13, 86, 87]
55	*K. duthia*	Distribution in China: Southwestern Sichuan, Northwestern Yunnan, Tibet;<br>Overseas distribution: Nepal, northern India to the Western Himalayas	Alpine shrub meadow at an altitude of 4100-4400 m	[13, 86, 90]
56	*K.capillifolia*	Distribution in China: Tianshan Mountains, Altai Mountains, Western Junggar Mountains, Kunlun Mountains, Pamirs, Inner Mongolia, Gansu, Qinghai, Xinjiang, Western and Southwestern Sichuan, Northwestern Yunnan, Tibet;<br>Overseas Distribution: Kazakhstan, Uzbekistan, Kyrgyzstan, Tajikistan, Turkmenistan, Pakistan, Western Mongolia, Afghanistan, Kashmir, Nepal	Alpine meadow, meadow steppe, thicket at an altitude of 2600-4600 m	[86]
57	*K. yadongensis*	Distribution in China: Southeast Tibet (Yadong)	Alpine meadows at an altitude of 4800 m	[13]

58	*K. tunicata*	Distribution in China: Northwest Yunnan (Yulong Mountain, Lijiang)	Alpine shrub meadow at an altitude of 3700-4250 m	[13]
59	*K. yushuensis*	Distribution in China: Tianshan Mountains, Altai Mountains, Western Junggar Mountains, Kunlun Mountains, Pamirs, Inner Mongolia, Gansu, Qinghai, Xinjiang, western and southwestern Sichuan, northwestern Yunnan, Tibet;<br>Overseas distribution: Kazakhstan, Uzbekistan, Kyrgyzstan, Tajikistan, Turkmenistan, Pakistan, Western Mongolia, Afghanistan, Kashmir, Nepal	Alpine meadows at an altitude of 3600-4600 m	[86]
60	*K. clarkeana*	Distribution in China: Southern Tibet;<br>Overseas distribution: Sikkim	Alpine oak forest at an altitude of 3000-3200 m	[86]
61	*K.longearistita*	Distribution in China: Western Sichuan (Rural Town Wuming Mountain)	Paddy meadow with river beach at an altitude of 3750 m	[86]
62	*K. myosuroides* subsp. *bistaminata*	Distribution in China: Gansu, Tibet, Xinjiang, Inner Mongolia, Qinghai, Ningxia, Sichuan	Alpine meadows, grassy slopes, inter-shrubs, 2100-4500 m altitude	[86, 87]
63	*K. woodii*	Distribution in China: Southern Tibet;<br>Overseas distribution: Bhutan	Grasslands, alpine meadows, 3300-4800 m altitude.	[86, 87]
64	*K. filicina* var. *filicina*	Distribution in China: Northwestern Yunnan and eastern Tibet	At an altitude of 2900-4000 m, under the forest or on the rocks by the river	[86]
65	*K. afilicina* var. *subfilicinoides*	Distribution in China: Northwestern Yunnan and eastern Tibet	At an altitude of 2900-4000 m, under the forest or on the rocks by the river	[86]
66	*K. vaginosa*	Distribution in China: Tibet, Yunnan<br>Overseas distribution: India (Sikkim), Nepal.	At 4000-4800 m, dry slopes, on rocks in alpine meadows.	[86]
